# Interaction standards for biophysics: anti-lysozyme nanobodies

**DOI:** 10.1007/s00249-021-01524-6

**Published:** 2021-04-11

**Authors:** Holly L. Birchenough, Hilda D. Ruiz Nivia, Thomas A. Jowitt

**Affiliations:** Wellcome Trust Centre for Cell Matrix Research, Faculty of Biology Medicine and Health, University of Manchester, Manchester, England

**Keywords:** Nanobodies, Molecular standards, Interaction technologies, Single domain antibodies, Phage display

## Abstract

There is a significant demand in the molecular biophysics community for robust standard samples. They are required by researchers, instrument developers and pharmaceutical companies for instrumental quality control, methodological development and in the design and validation of devices, diagnostics and instrumentation. To-date there has been no clear consensus on the need and type of standards that should be available and different research groups and instrument manufacturers use different standard systems which significantly hinders comparative analysis. One of the major objectives of the Association of Resources for Biophysical Research in Europe (ARBRE) is to establish a common set of standard samples that can be used throughout the biophysics community and instrument developers. A survey was circulated among ARBRE members to ascertain the requirements of laboratories when using standard systems and the results are documented in this article. In summary, the major requirements are protein samples which are cheap, relatively small, stable and have different binding strengths. We have developed a panel of sdAb’s or ‘nanobodies’ against hen-egg white lysozyme with different binding strengths and suitable stability characteristics. Here we show the results of the survey, the selection procedure, validation and final selection of a panel of nanobody interaction standards.

**Supplementary Information:**

The online version contains supplementary material available at 10.1007/s00249-021-01524-6.

## Introduction

There is an unmet need within the molecular biophysics community for samples that can be used as standards across multiple diverse biophysical instruments. There are many different samples that are utilized as standards within individual laboratories or provided by instrument manufacturers but there is a lack of continuity and consistency significantly impacting instrumental design, methodological development and data equivalence across multiple techniques. Standards are immensely important in the development of instrumentation and methodology and the availability of common standards will allow cross validation of techniques. Currently developers of biophysical devices use a variety of different standard samples for instrument validation and therefore comparability between instruments is unclear for both developers and customers. Likewise, research labs require standards for training, development and validating instrument performance. Benchmarking of instruments and methods is a critical way of ensuring instrument performance and highlighting common user errors. The development of common standard operating procedures (SOP’s) using well-studied standards would be beneficial for both biophysics resource labs and instrument developers alike.

ARBRE is a network of biophysics facilities and resource infrastructures that is ideally placed to spearhead the development of common standard samples that can be implemented in cross-instrumentation comparisons and benchmarking studies and in the development of SOP’s for users across the biophysics communities. As part of an ARBRE supported initiative to develop standards, we disseminated a questionnaire to ascertain the most desirable qualities of standard samples prior to the development of a set of standard protein samples focusing on interaction analysis. The full results of the survey are presented in the supplemental material (Sup. 1 and 2). The standards we developed are single domain antibody (sdAb) molecules with hen egg white lysozyme (HEWL) as the antigen or binding partner. Single domain antibody fragments often referred to as nanobodies, which is a trademark of Ablynx, are based on the variable domain of heavy chain-only camelid antibodies (*V*_HH_) (Hassanzadeh-Ghassabeh et al. 2013; Jovcevska and Muyldermans 2020; Muyldermans 2020; Revets et al. 2005; Vu et al. 1997). These small protein domains of between 12 and 15 kDa are highly stable and easy to produce in large quantities, very soluble (Kim et al. 2014; Rouet et al. 2015; Salema and Fernandez 2013; Vu et al. 1997) and are also easy to be modified with functional groups, protein adjuncts and fluorescent probes (Al-Baradie 2020; Carrington et al. 2019; Jovcevska and Muyldermans 2020; Mortensen et al. 2020; Wang et al. 2019; Wu et al. 2018; Yu et al. 2020). The paratope of *V*_HH_ sdAb’s consists of three variable complementarity determining regions (CDR) 1, 2 and 3 which convey ligand specificity and binding strength. Typically, CDR3 confers the greatest variability and is primarily responsible for ligand binding and specificity (McMahon et al. 2018; Moutel et al. 2016; Rouet et al. 2015; Zavrtanik et al. 2018). There are several reports on sdAb structure and stability (Kim et al. 2014; Muyldermans 2020; Zavrtanik et al. 2018), which show that the CDR3 loop is also primarily responsible for the camelid *V*_HH_ monomeric nature as compared to the human equivalent which has a propensity to dimerize through the *V*_H_–*V*_L_ interface, which is protected by the camelid CDR3 loop. Historically, nanobodies were generated in a similar way to full length antibodies through immunization of llamas with the antigen of choice. However, there are now many synthetic libraries using ribosomal, phage or yeast display systems which have common frameworks but with variable CDR’s (Kajiwara et al. 2020; McMahon et al. 2018; Moutel et al. 2016; Zimmermann et al. 2020).

The ability to generate multiple binding strength molecules against a single target is one of the primary reasons that sdAb molecules such as nanobodies are extremely attractive as molecules for standards. We have used both site-directed mutagenesis and phage display to generate a selection of nanobody molecules which are raised against HEWL and assessed their suitability for standard samples.

## Experimental

### Nanobody expression

The nanobody *V*_HH_ sequence was engineered into pET-22B expression vector with a C-terminal 6-His tag. The vector was transformed into competent T7-Express *E. coli* cells (New England Biolabs) and selected on 100 µg/ml ampicillin plates. One colony was selected for overnight growth in 5 ml LB broth supplemented with 100 µg/ml ampicillin shaking at 37 °C. The cells were pelleted by centrifugation at 2000 rpm for 5 min and resuspended in 5 ml of sterile LB. This suspension was used to inoculate 1 l of Magic Media™ (Thermofisher) divided between two 2 l baffled flasks and cells were incubated for 24 h at 28 °C on a rotary shaker set to 180 rpm. Cells were extracted by centrifugation at 4500 rpm for 20 min at 4 °C with 250 ml cell suspension per 500 ml centrifuge tube and resuspended in 50 ml 50 mM phosphate-buffered saline (10 mM phosphate buffer with 138 mM NaCl, 2.7 mM KCl) with 1% glycerol pH 7.0. Cells were then frozen at -80 °C until needed. Protein extraction: cells were thawed quickly then left on ice for 20 min before sonication in a Soniprep 150 tissue homogenizer (4 × 15 s) kept on ice. Cell debris was centrifuged at 14,000 rpm at 4 °C for 10 min and the supernatant collected. The supernatant was diluted 1:2 in 10 mM PBS pH 7.4 and injected onto a 5 ml Profinity IMAC column (BioRad) at 4 ml/min using a BioRad NGC FPLC. Protein was eluted in PBS supplemented with 0.5 M ultrapure imidazole (Sigma) without a gradient and collected in deep-well 96-well plates. The elution peak was collected and further purified on a 10/300 Superdex-75 column in PBS plus 0.005% P20 with a flow-rate of 0.75 ml/min.

### Surface plasmon resonance (SPR)

SPR was performed using a Biacore T200 (Cytiva life science). Lysozyme was immobilized to a maximum loading level of 100 units using EDC/NHS coupling chemistry to link lysozyme primary amines to carboxyl groups on a CM5 carboxymethyldextran sensor chip. Unused activated carboxyl groups were blocked using ethanolamine. A blank sensor channel was activated and blocked in the same manner, but with no lysozyme attached. Lysozyme contains three surface lysine residues, none of which are within the nanobody binding pocket. Kinetic titrations were performed in PBS containing 0.01% tween20 using the multi-cycle kinetics wizard and a concentration series-dependent on the nanobody but typically from between 100 and 200 nM. Regeneration was perfomed by sequential 5 s injections of 100 mM Glycine pH 2.2 and 10 mM NaOH at 30 μl/min. Data are reported as an average of between 2 and 4 replicates and all fitting is performed using a Langmuir 1:1 binding model within the BiaEvaluation software.

### Isothermal titration calorimetry (ITC)

ITC was performed using a Malvern PEAQ-ITC. Samples were purified from the gel-filtration column in phosphate buffered saline pH 7.4 (PBS) and frozen in 1 mg/ml (~ 71 µM). For ITC, samples were defrosted on ice and diluted to a concentration of 20 µM for H04, H106A and Cab-Lys3 and the rest were diluted to 30 µM in PBS containing 0.005% tween 20. HEWL lysozyme powder (Roche > 95%) was made to 10 mg/ml (~ 690 µM) in PBS plus 0.005% tween 20 and further diluted to 200 µM for H04/H106A or 300 µM for the rest of the nanobodies. Lysozyme was equilibrated in the cell and nanobody was titrated using a preliminary injection of 0.2 µl, followed by 18 succesive 2 µl injections with a reference power of 10, a stirring speed of 750 rpm and a spacing of 120 s between injections. Samples were run in triplicate and fitted using a single site model following subtraction.

### Size exclusion calorimetry with multi-angle light scattering

Samples (0.5 ml) were loaded onto a Superdex-75 10/300 (GE) column running at a flow rate of 0.75 ml/min in PBS (pH 7.4). Samples eluting from the column passed through a Wyatt Helios 18-angle laser photometer with the 13th detector replaced with a Wyatt QELS detector for the simultaneous measurement of hydrodynamic radius. This was coupled to a Wyatt Optilab TrEX refractive index detector and the hydrodynamic radius, molecular mass moments and concentrations of the resulting peaks were analyzed using Astra V6.

## Results and discussion

### Survey results

**Fig. 1:**
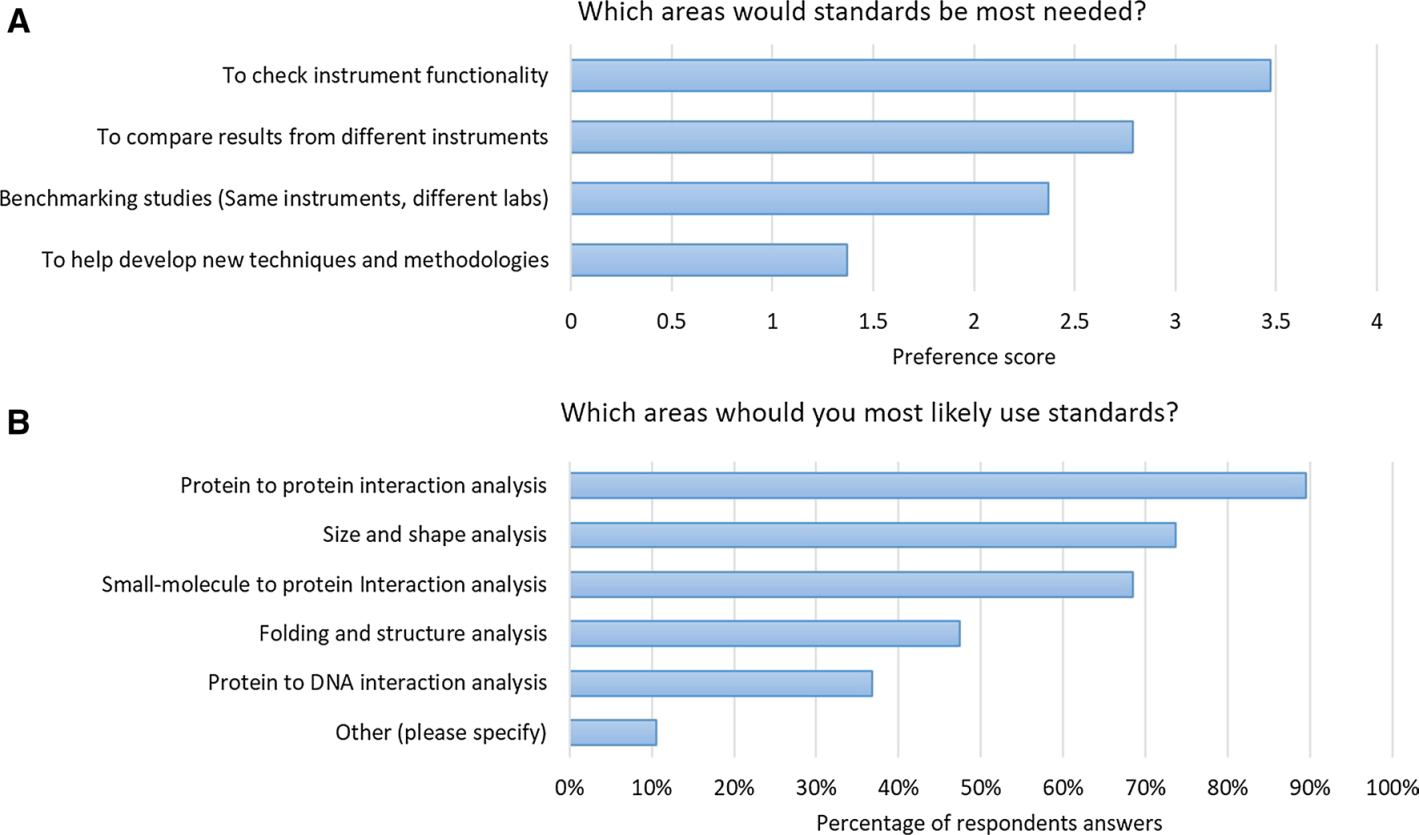
Results of survey questions. **a** Which areas would standards be most needed? **b** Which areas would you most likely use standards?

A survey was distributed to the ARBRE mailing-list and was completed by 19 anonymized biophysics laboratories across Europe. The full survey results can be found in the supplemental information. Figure 1 shows the results of two questions: which areas of biophysics would you use standard samples, and which areas of biophysics would standards be most needed?

**Fig. 2:**
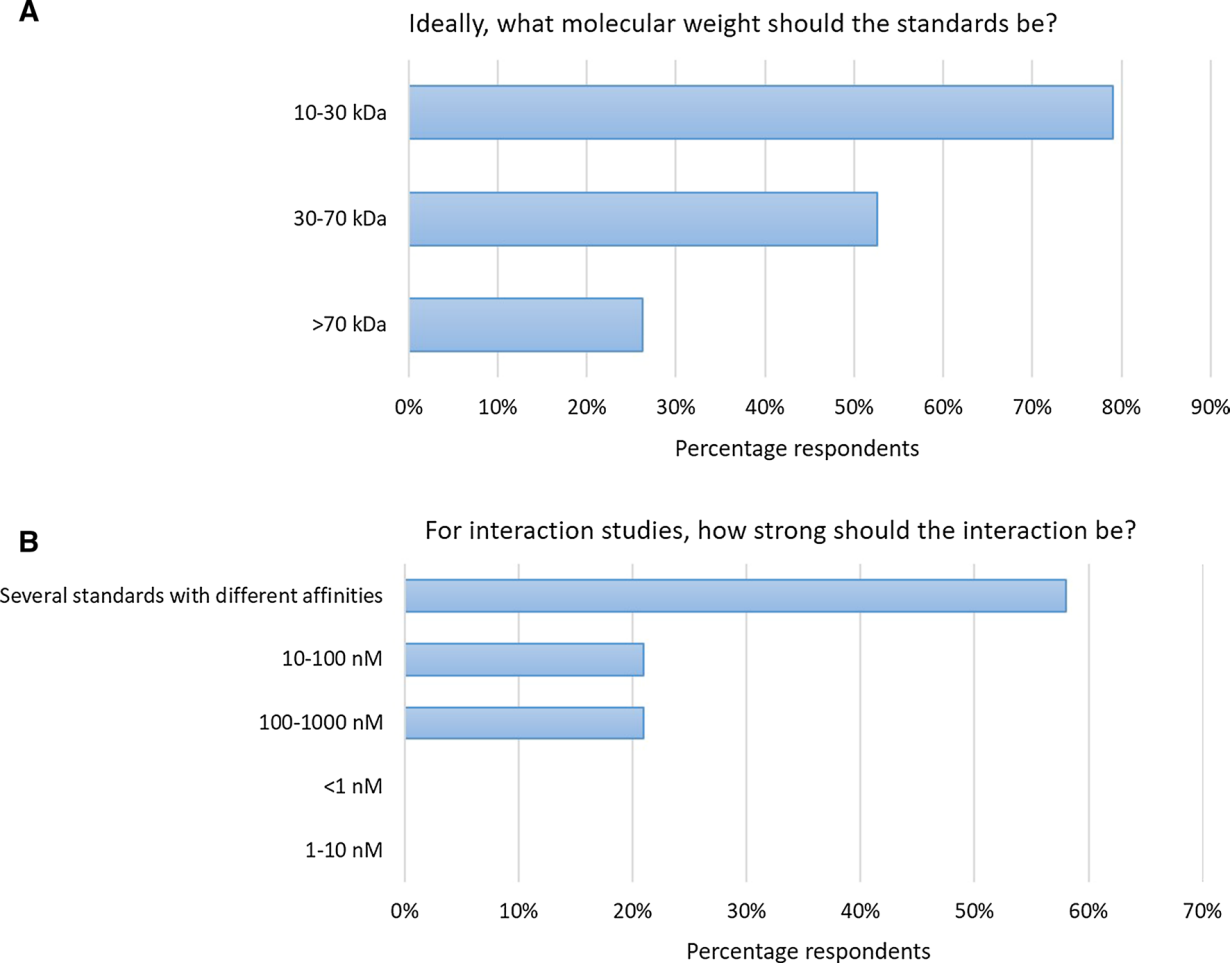
Survey questions relating to **a** what molecular weight standards should ideally be? And **b** how strong should the interaction be?

First, the questions determine what the standards would be used for, for example in testing their instruments for conformity or creating new methods. It also then checks which areas of biophysics the respondents think they need the standards the most, for example in interaction analysis or structural biophysics, and whether they prefer protein standards to DNA or small molecules. Of the biophysics labs that responded, the majority would like to use standards for testing their own equipment and comparing the results with other instrumentation. This is followed by a desire for cross lab comparisons of the same instrument or benchmarking. Given that most labs wish to check their own instruments conformity, benchmarking is presumably to test that their own instruments are performing to the best of their abilities compared to other labs. Most labs prefer protein–protein interaction standards rather than protein–DNA or protein–small molecule as protein–protein interaction analysis is probably a more common practice. Interestingly there is a large number of respondents who would like to have hydrodynamic standards. There is a clear need for both interaction and hydrodynamic standards with the majority of respondents indicating that they do not have in-house standards to use. The majority of labs required standards for interaction technologies such as surface plasmon resonance (SPR) and isothermal titration calorimetry (ITC) although it could be that the respondents of the survey were more used to these technologies. Focusing on interaction techniques, Fig. 2 shows the results for two practical questions: What would be the ideal molecular weight of standard samples, and what would be the ideal binding strengths?

Because molecular biophysics encompasses a wide variety of instrumentation, a single standard may not be appropriate. The results show that molecules with a molecular weight of between 10 and 30 kDa would be ideal. Binding strengths are also very important in the selection process for standard samples. Some instrumentation cannot investigate very tight binding, for example ITC cannot easily measure *K*_D_’s below 5 nM unless indirect approaches are used such as ligand displacement which would not be suited for a standard sample. Likewise some techniques cannot measure very weak interactions. The results to this question were very clearly showing that there is not one standard that will fit all purposes. There is a need for several standards with different binding strengths, although no respondents required standards that had a binding strength tighter than 1–10 nM, or weaker than 1 µM.

### Rationale behind the development of nanobodies as standards

**Fig. 3:**
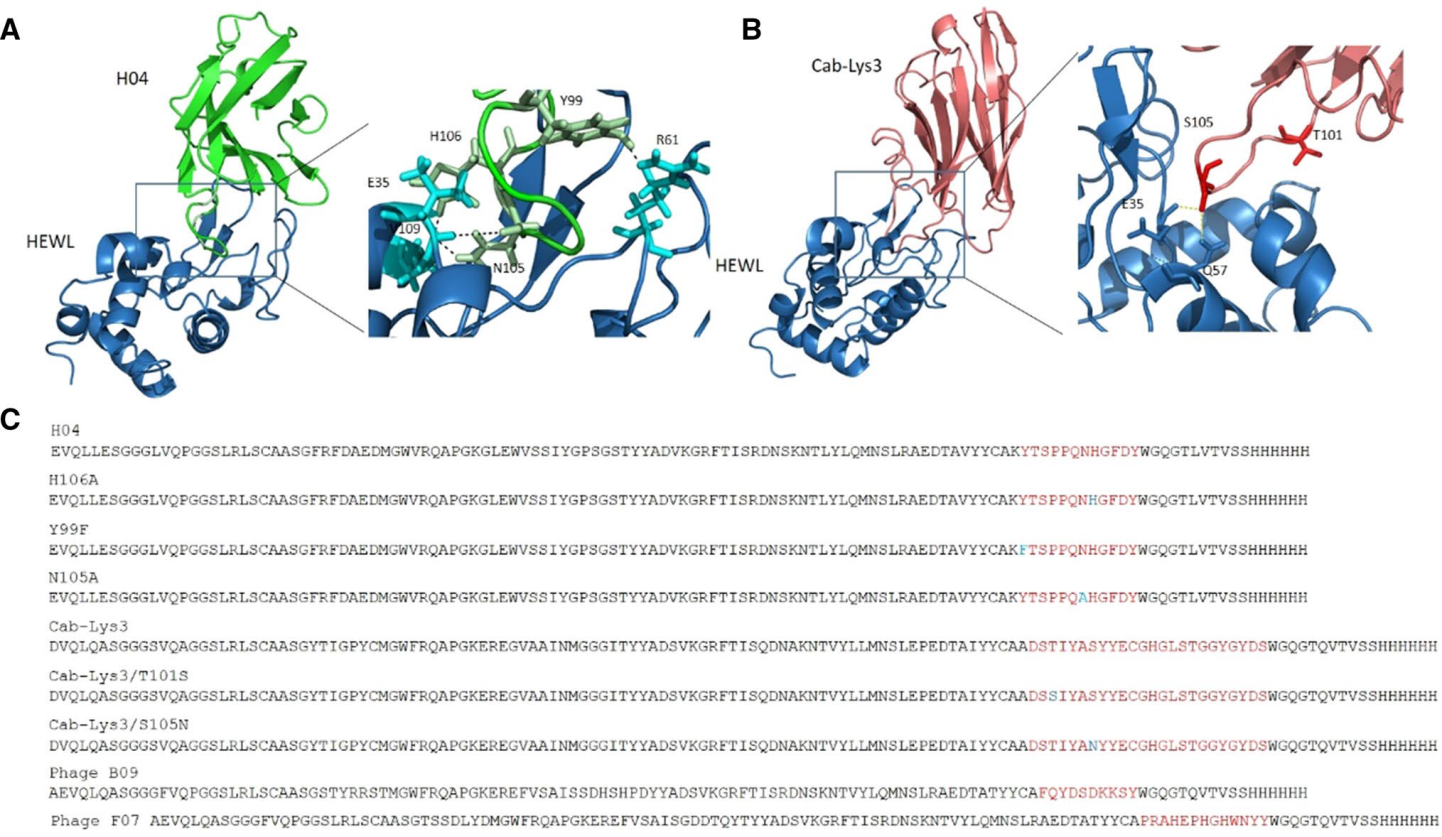
**a** H04 bound to lysozyme highlighting CDR3 residues H106, Y99, N105 and **b**, Cab-Lys3 highlighting CDR3 residues S105 and T101. **c** Sequences of nanobodies with suitable characteristics for use as standard proteins. The red letters highlight the CDR3 loops and mutated amino acids are highlighted in blue

The criteria for gold-standard interaction standards therefore are; cheap, stable, 10–30 kDa with multiple binding strengths and well-characterized. To fulfil these criteria, we decided to investigate the applicability of nanobody molecules raised against lysozyme. Nanobodies typically have been shown to have high functional stability over a large pH range, good colloidal properties across different buffers and are easy to produce in high yields (Kim et al. 2014; Rouet et al. 2015; Salema and Fernandez 2013; Vincke et al. 2009). They have also been shown to be amenable to downstream engineering such as Fc fusion, GFP, double specificity, etc. (Marturano et al. 2020; Mortensen et al. 2020; Wu et al. 2018; Yang and Shah 2020). These attributes therefore make nanobodies a good choice to investigate their potential as biophysics standards. The target we decided to use was hen egg white lysozyme (HEWL), and for the purposes of this article will be called lysozyme. Lysozyme is a small monomeric 14.5 kDa molecule that is extensively characterized, stable, cheap and easy to purchase. Lysozyme has a deep cleft which is the native carbohydrate-binding region (Fig. 3) which makes it an ideal target for the CDR3 loop of nanobodies.

### Nanobody standard development

The challenge for creating standards is to maintain a common structure, but to have different binding strengths. Most commercial nanobodies are very tight binders and one of the challenges for us was to create a selection of weaker binders. Mutation of the CDR3 loops is one way we created weaker binding molecules as well as to isolate weak binders from phage display. Several groups have previously made nanobodies to lysozyme (Dumoulin et al. 2002; Guardiola et al. 2020; Muyldermans and Lauwereys 1999; Rouet et al. 2015; Vincke et al. 2009; Vu et al. 1997) but the focus of many of these was to create nanobodies with high binding strengths. Indeed, the selection process in phage display naturally favors selection of tighter binders. We decided to investigate two of these nanonodies, Cab-Lys3 (crystal structure 1XFP) developed by Muyldermans and Lauwereys (Muyldermans and Lauwereys 1999) and H04 developed in Daniel Christ’s group (Rouet et al. 2015) (Fig. 3). With the help of the crystal structure to H04 (PDB code 4U3X), we first mutated residues Y99 to a phenylalanine, N105 and H106 to alanine which disrupts hydrogen bonds with residues R61, V109 and E35, respectively, in the carbohydrate-binding cleft of lysozyme. We speculated that these mutations would significantly decrease the binding strength.

**Fig. 4:**
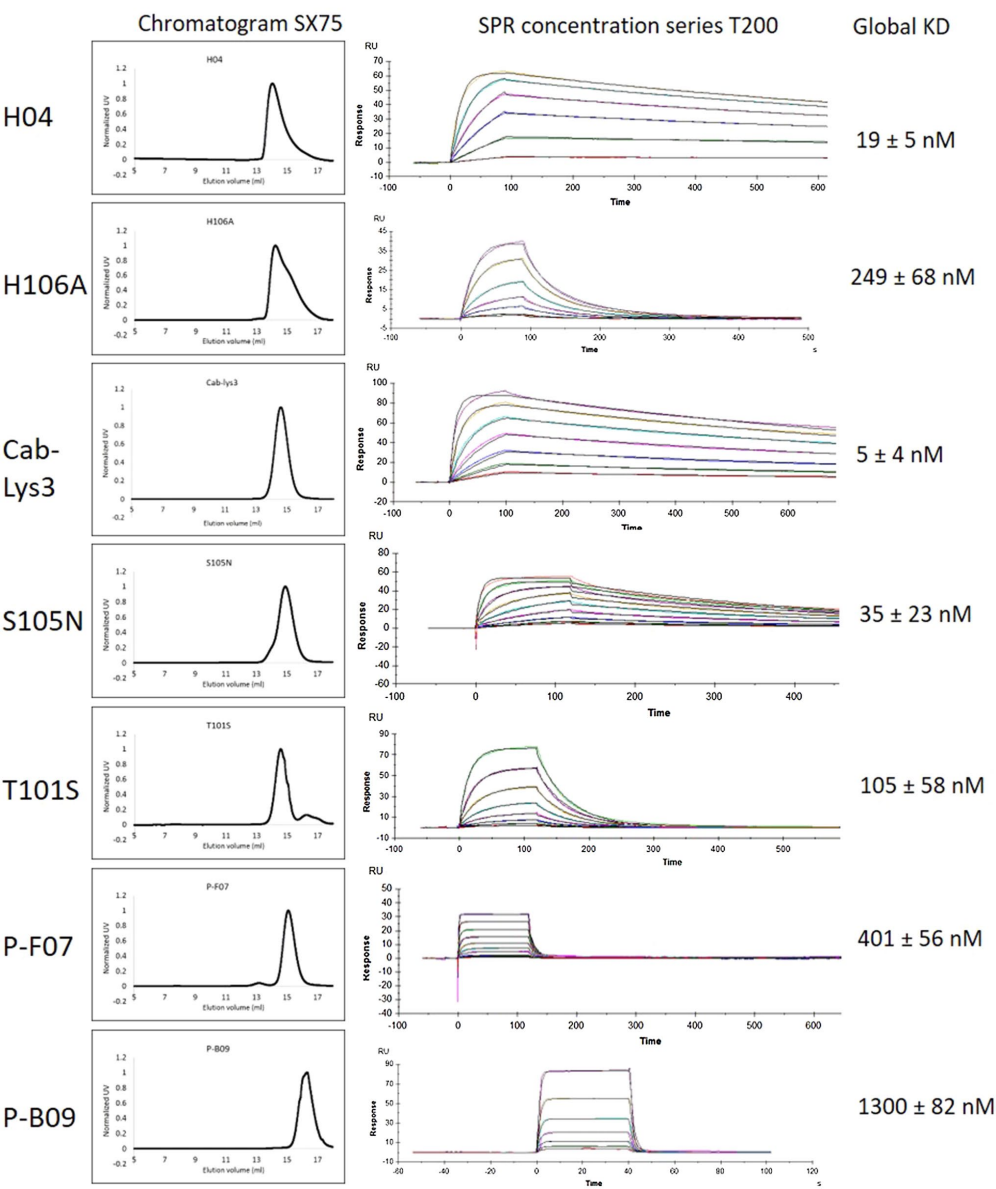
Chromatograms from a Superdex-75™ gel filtration column following = g purification and concentration series binding experiments using SPR (Biacore T200) fitted with a global 1:1 non-linear model and the affinity constant for each as determined by the model

The mutation Y99F and N105A which shares hydrogen bonds with Valine 109 and glutamic acid 57 completely abolished binding, therefore, these candidates were not taken forward. H106A, however, lowered the binding strength to ~ 250 nM by surface plasmon resonance (SPR) (Fig. 4). Based on the work by De Genst et al*.* (2002) we mutated residues within the CDR3 loop of Cab-Lys3, T101 to a serine and S105 to an asparagine. Cab-Lys3 has an extremely long CDR3 binding loop of 24 residues compared to H04 which has 12 residues. Typically CDR3 loops range between 9 and 18 residues (Moutel et al. 2016). This very long binding loop has an extra intraloop disulphide bridge which has been postulated to confer extra stability and increase binding affinity (Dumoulin et al. 2002; Kunz et al. 2018; Mendoza et al. 2020). The WT Cab-Lys3 in our hands has a binding strength of ~ 5 nM by SPR which is far too tight for many techniques. The two mutations T101S and S105N produced molecules with SPR affinities of 105 and 35 nM, respectively (Fig. 4).

Using phage display, we generated several nanobody molecules that have lower binding strengths. Molecules were generated using the NaLi-H1 synthetic library (Moutel et al. 2016) (Hybrigenics, Paris) which gave several molecules that were suitable, namely Phage-F07 and Phage-B09. These molecules have affinities based on SPR of 400 and 1300 nM, respectively (Fig. 4).

### Suitability of the samples as standards

Standard samples need to be cheap to purchase, meaning they require an ease of manufacture. We assessed the purification levels of each of the clones (Table 1) expressed in pET-22B with a 6-His tag for purification. Purification was a simple two-step His-NTA followed by Superdex-75 gel filtration in PBS (see methods). Expression yields ranged ~ three–fivefold, Cab-Lys3 constructs with the lowest expression at ~ 10 mg/l, and H04/H106A expressing up to 50 mg/l. The phage display clones express at between 20 and 30 mg/l depending on the clone. P-F07 and P-B09 were ~ 30 mg/l. Whilst there is a significant difference, all these expression levels are suitable for standard sample production.

**Table 1:** Expression levels and binding data for the nanobody standards

Nanobody biophysical data								
Nanobody	Expression (mg/l)	SPR *K*_D_ (nM)	SPR *k*_a_ (M s^−1^) × 10^4^	SPR *k*_d_ (s^−1^) × 10^–2^	ITC *K*_D_ (nM)	Δ*H* (kcal/mol)	Δ*G* (kcal/mol)	− *T*Δ*S* (kcal/mol)
H04 20 °C	50	19 ± 5	4.3	008	24 ± 3	− 17.6	− 10.4	7.2
H106A 20 °C	50	124 ± 68	45	5.6	296 ± 56	− 17.0	− 9.3	7.7
Cab-Lys3 20 °C	10	5 ± 4	4.1	0.09	27 ± 16	1.6	− 9.8	− 11.6
Cab-Lys3 37 °C	10	ND			5 ± 3	− 4.02	− 11.7	− 7.73
S105N 20 °C	10	35 ± 23	7.9	0.28	125 ± 66	2.6	− 9.3	− 11.6
S105N 37 °C	10	ND			73 ± 22	− 3.3	− 10.1	− 6.9
T101S 20 °C	10	135 ± 35	16	2.9	113 ± 39	3.0	− 9.3	− 12.4
T101S 37 °C	10	ND			105 ± 15	− 5.1	− 9.9	− 4.7
P-F07 20 °C	30	401 ± 56	2	8.2	685 ± 92	− 23.5	− 8.4	15.1
P-B09 20 °C	30	1300 ± 82	45	60	1430 ± 113	− 14.5	− 8.1	6.4

Errors are from triplicate measurements

*ND* not determined

**Fig. 5:**
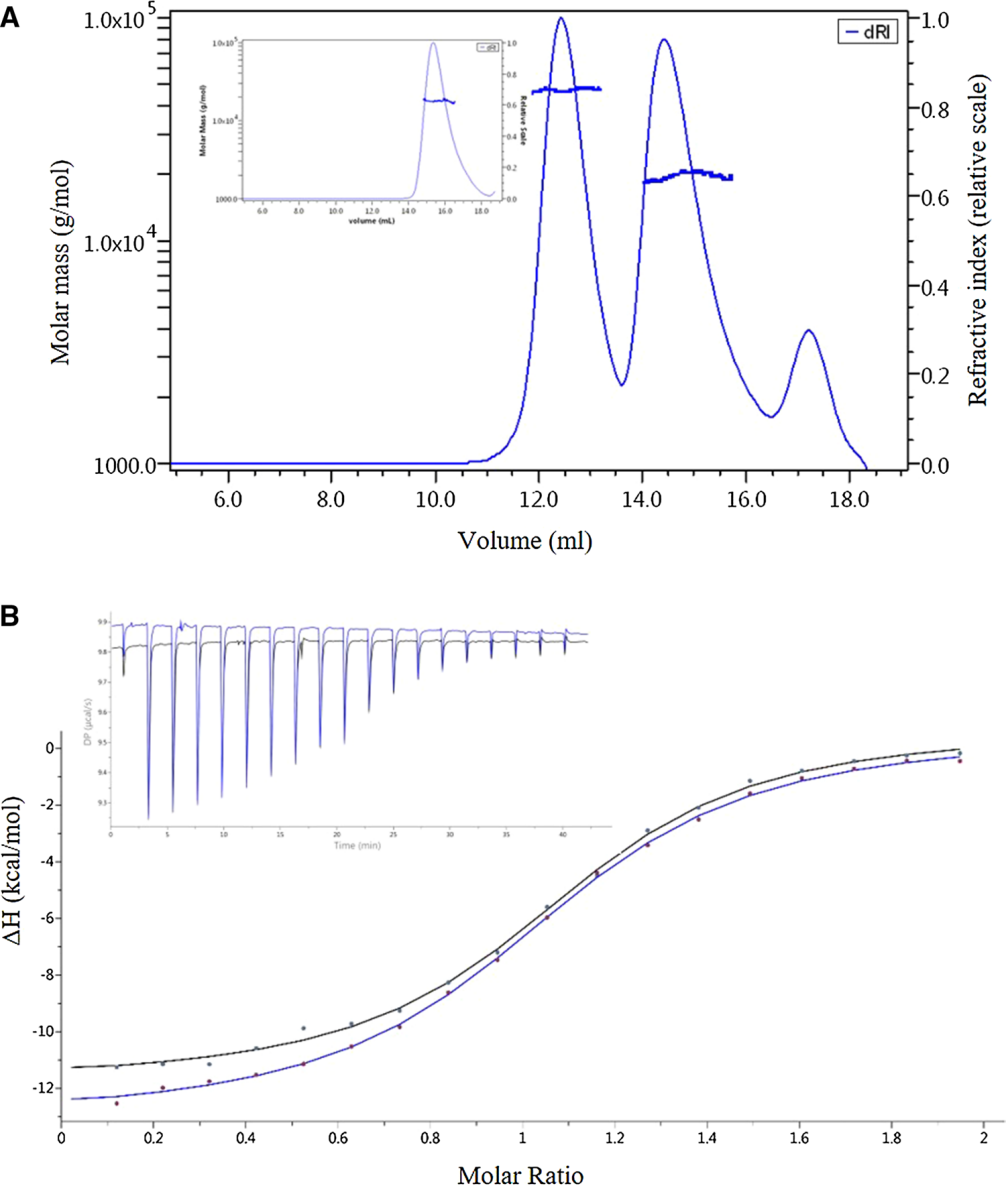
**a** Multi-angle light scattering of H04 eluting from a superdex-75 gel filtration column during the purification process. The non-symmetrical monomeric peak from the gel filtration column elutes at 15 ml and dimers elute earlier at 12.5 ml. Monomers and dimers encountered during purification are non-reversible and can be separated (inset). **b** Isothermal titration calorimetry of P-B09 6 months apart at 4 °C. Black trace is fresh P-B09, and the blue trace is 6 months later. Raw ITC (inset) and isotherm similarities highlights the stability and repeatability of the molecules

Samples also need to be monomeric and stable. Figure 4 shows the elution positions of the nanobodies on a Superdex-75 gel filtration column following purification. Monomeric nanobodies elute at ~ 15 ml. However, with the H04 and H106A variants we observed non-symmetrical peaks. This broader peak has a consistent mass across the different eluting species (as shown using MALS (Fig. 5) which suggests that there is a proportion of the population that interacts with the column matrix.

Purification of all the nanobodies produces some dimers that are eluted from the His-trap column and are clearly visible by gel filtration. These dimers are more apparent with H04 and H106A and are non-reversible, which can be shown by gel filtration (Fig. 5a, inset) and by analytical ultracentrifugation (data not shown). Dimers are unreactive to lysozyme and for this reason, any dimers present in the samples cannot be seen using SPR and has no effect on the kinetics. Any dimers present in the sample does; however, have an effect on the *n-*number using ITC. Interestingly, we found that repeated freeze–thaw cycles to be the main contributing factor for a decrease in *n*-numbers indicating that freeze–thaw cycles contribute to nanobody aggregation. We also repeated ITC experiments with P-B09 6 months apart (proteins kept in sterile PBS) (Fig. 5b). There was little difference in the *n-*values and near identical results which suggests that the proteins are extremely stable when kept at 4 °C. Taken together the results indicate that these nanobodies represent a very stable framework which can be produced in large quantities to keep prospective costs to a minimum. They are amenable to multiple interaction technologies and we have produced molecules with several different affinities.

### The H04 and phage-display ***V***_HH_ sdAb’s exhibit different binding modes of action to the Cab-Lys3 mutants

**Fig. 6:**
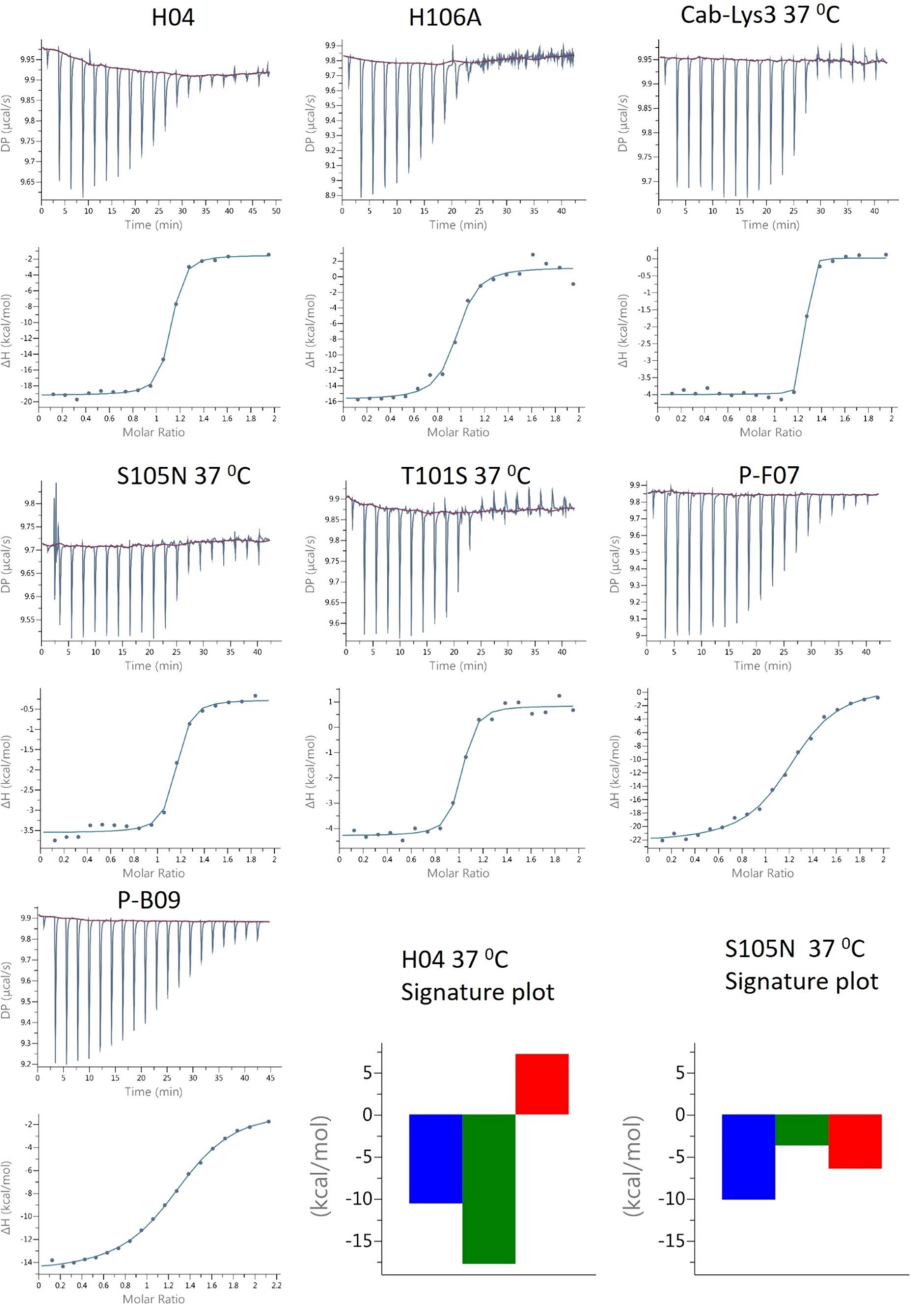
ITC thermograms of the nanobody constructs. The signature plots of H04 and S105N are shown which highlights the significant difference in the mode of action between the two nanobodies

All the molecules in this report exhibits near ideal 1:1 SPR traces (Fig. 4), but the Cab-Lys3 framework sdAbs have very different ITC characteristics as shown by ITC (Fig. 6). At 20 °C all the molecules fit to a Langmuir 1:1 binding model using SPR, but at 20 °C Cab-Lys3, T101S and S105N have a weak endothermic response in contrast to H04, H106A, P-F07 and P-B09 which have strong exothermic binding responses. This is true at both 25° and 37 °C. This is very interesting as these molecules both bind within the HEWL carbohydrate-binding cleft. The major difference between the two molecules is the length of the CDR3 loop. Cab-Lys3 has a very long 24-amino acid loop with an extra disulphide link between additional cysteines in CDR1 and CDR3. At room temperature the shorter CDR3 loop sdAb’s produce a large exothermic response with van’t Hoff enthalpy (Δ*H*) values in the range of − 14 to − 23 kcal/mol (Table 1), and this does not change significantly with increasing the temperature (data not shown).

Conversely Cab-Lys3, T101S and S105N have small positive Δ*H* at room temperature and a large negative entropic effect upon binding to lysozyme which seems to suggest that the long binding loop requires significant reorganization to fit in to the binding cleft. The binding becomes more favorable by increasing the temperature, decreasing the enthalpy and increasing the entropy. This seems to suggest that there is a enthalpy–entropy compensation mechanism ocuring (Chodera and Mobley 2013) which does not occur with the other nanobodies in this study. This system could be used as standard model for entropy–enthalpy compensation although this would need to be studied in greater detail.

## Conclusions

The results of the survey into the need for standards in biophysics suggested the community requires standards for interaction analysis which are protein–protein based, between 10 and 30 kDa, stable, well-characterized and of several different affinities. We have developed a sdAb *V*_HH_ system to lysozyme which fulfils these requirements and has been used successfully in a benchmarking study for microscale thermophoresis. We have shown that nanobodies can be extremely stable but they need to be carefully selected, and it is possible to manipulate them to alter the binding strength to create molecules with the desired binding affinities. We have generated a panel of nanobodies that have affinities to lysozyme of between 5 and 1300 nM which are amenable to multiple techniques and we have also shown that some of these nanobodies have interesting thermodynamics which increases the usability of these nanobodies for testing equipment. It is important to note that nanobodies require careful selection to eliminate those molecules with undesirable characteristics such as aggregation and there is not one nanobody that is suitable for all techniques. For example, at room temperature the Cab-Lys3 molecules are not ideal for ITC. However, these nanobodies represent an exciting possibility to develop standard operating procedures that are adopted by labs and instrument developers alike and to standardize the way instrumental biophysics users are trained. They will hopefully allow manufacturers to use the same systems to develop their instruments and for users to benchmark their instruments and methodological developments.

## Supplementary Information

Below is the link to the electronic supplementary material.

Supplementary file1 (DOCX 1345 KB)
