# Supplementary material for: Interaction standards for biophysics: anti-lysozyme nanobodies
Source: Eur Biophys J. 2021 Apr 11;50(3-4):333–43. doi: 10.1007/s00249-021-01524-6 (PMC8189969; doi:10.1007/s00249-021-01524-6)
Supplement: Supplementary file 1 — Supplementary file1 (DOCX 1345 KB) [file 249_2021_1524_MOESM1_ESM.docx]

**Supplemental figures**


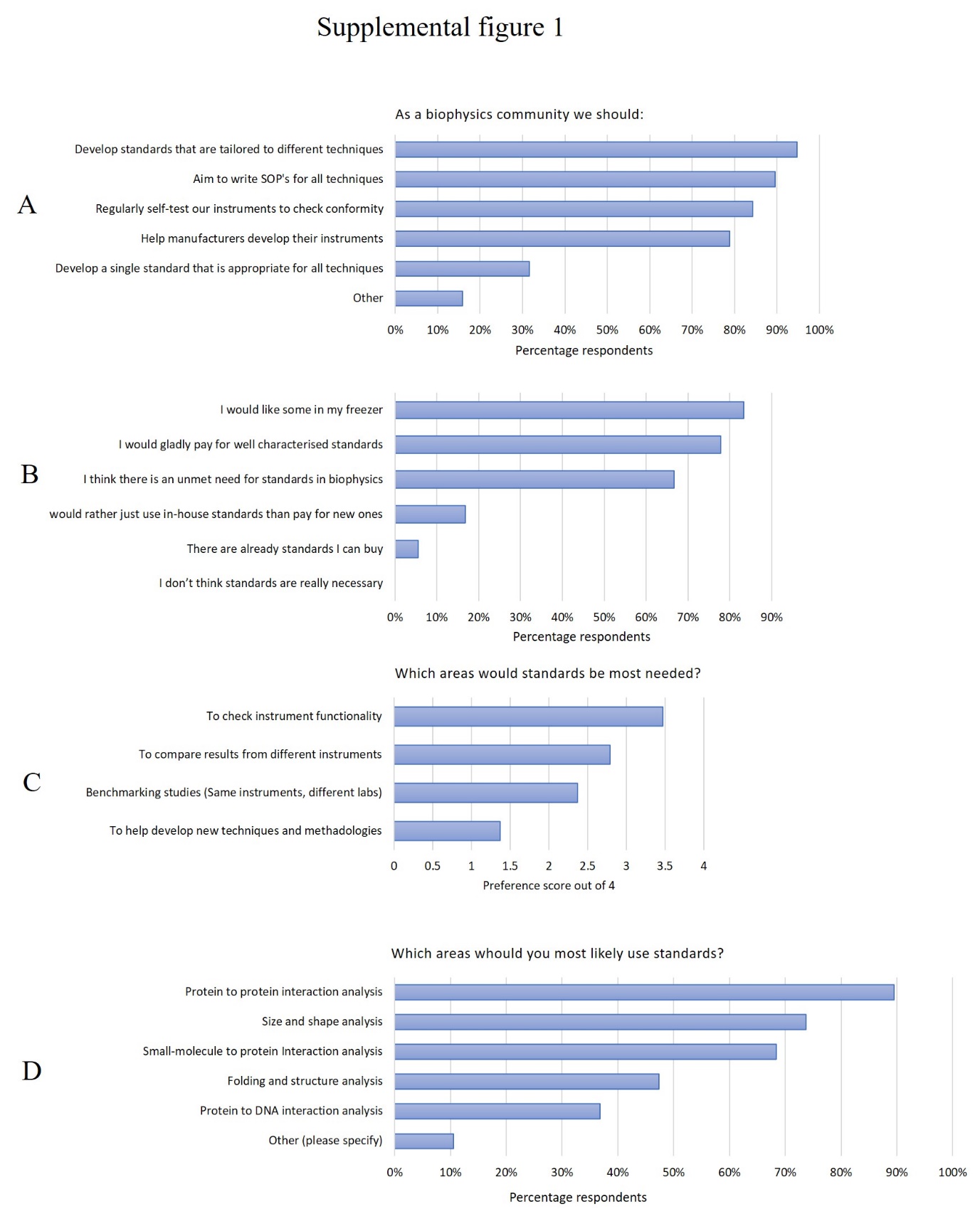


Supplemental figure 1: Survey regarding the requirements for standards questions A to D.


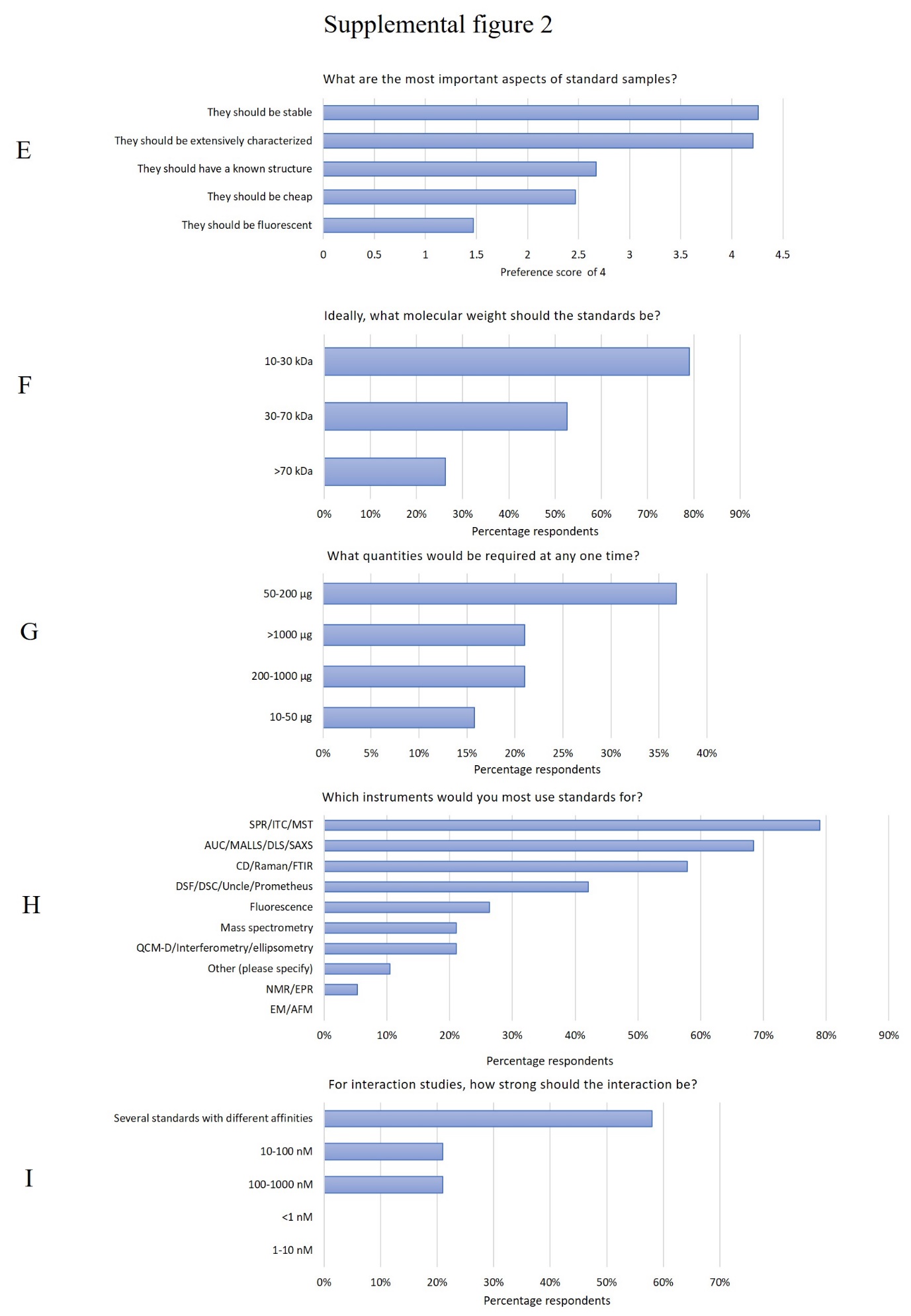


Supplemental figure 2: Survey regarding the requirements for standards questions E to I.
